# Cuterebra Emasculator in Dog

**Published:** 1893-12

**Authors:** Cecil French

**Affiliations:** Secretary-Treasurer Montreal Vet. Med Association


### CUTEREBRA EMASCULATOR IN DOG.

By Cecil French.

*Secretary-Treasurer Montreal Vet. Med. Association.*

I beg to inform you of the occurrence of the larva of a botfly in the testicle of the dog. It was extracted from the scrotum at the College clinic here. I forwarded it to Prof. Fletcher of the Experimental Farm at Ottawa, and he pronounced it to belong to the Genus Cuterebra, individuals of which are known to infest squirrels, hares and mice, but he had never heard of a dog being previously affected.

An article on "Cuterebra Emasculator" appears in the U. S. Bulletin, "Insect Life," for January, 1889.

The dog was apparently indifferent to the fact that the parasite was slowly emasculating him.

